# Cellular uptake and cell-to-cell transfer of polyelectrolyte microcapsules within a triple co-culture system representing parts of the respiratory tract

**DOI:** 10.1088/1468-6996/16/3/034608

**Published:** 2015-06-04

**Authors:** Dagmar A Kuhn, Raimo Hartmann, Kleanthis Fytianos, Alke Petri-Fink, Barbara Rothen-Rutishauser, Wolfgang J Parak

**Affiliations:** 1 Adolphe Merkle Institute, Université de Fribourg, Fribourg, Switzerland; 2 Department of Physics, Philipps Universität Marburg, Marburg, Germany; 3 CIC Biomagune, San Sebastian, Spain

**Keywords:** microcapsules, triple co-culture model, uptake, transfer, respiratory tract

## Abstract

Polyelectrolyte multilayer microcapsules around 3.4 micrometers in diameter were added to epithelial cells, monocyte-derived macrophages, and dendritic cells *in vitro* and their uptake kinetics were quantified. All three cell types were combined in a triple co-culture model, mimicking the human epithelial alveolar barrier. Hereby, macrophages were separated in a three-dimensional model from dendritic cells by a monolayer of epithelial cells. While passing of small nanoparticles has been demonstrated from macrophages to dendritic cells across the epithelial barrier in previous studies, for the micrometer-sized capsules, this process could not be observed in a significant amount. Thus, this barrier is a limiting factor for cell-to-cell transfer of micrometer-sized particles.

## 1. Introduction

The inhalation pathway is a promising entry portal for drug delivery. Due to the characteristics of the lung, e.g., huge internal surface of the lung parenchyma (i.e., alveoli and airways) of about 150 m^2^ and millions of immune cells [1, 2], the uptake of particulate materials is favored. Hereby, the transfer from air to liquid, e.g., of particles, is controlled by structural as well as by cellular barriers, i.e., the human epithelial alveolar tissue barrier [3]. This barrier constitutes a complex system involving the interplay of several different types of cells. Still, essential features of the human epithelial alveolar barrier can be simulated with cellular model systems. A well-characterized three-dimensional (3D) model of this barrier has been established, which is composed of epithelial cells, human blood monocyte-derived macrophages (MDMs), and dendritic cells (MDDCs) [3]. With triple cell co-cultures (TCCs), it has been demonstrated, that nanoparticles, in particular polystyrene particles with diameters of 0.1–1 *μ*m, can be transferred across the lung barrier by being passed from macrophages to dendritic cells [4]. While this process has been reported in several publications [4, 5], so far it has remained unclear to which range of particle sizes this process applies. Potentially such a process could be harnessed for particle-based drug delivery applications. Particles in general are endocytosed by macrophages. Thus, targeting the dendritic cells located underneath the epithelium could be achieved by active passing particles from macrophages across the epithelium to the dendritic cells.

We wanted to probe this concept for polyelectrolyte microcapsules (PEMs) [6–9], which are a universal delivery system. PEMs are fabricated by layer-by-layer assembly of charged polyelectrolytes around sacrificial templates [9] and can be readily used to encapsulate molecular cargo [10, 11]. Uptake of PEMs by cell lines and release of encapsulated cargo has been demonstrated with two-dimensional (2D) cell cultures [12–14]. While interaction of PEMs with cell lines is relatively well studied [15, 16], also involving the investigation of internalization pathways and cytotoxic effects [17, 18], most of these studies are based on 2D *in vitro* models. While there are several reports about *in vivo* applications, such as vaccination [19], the behavior of cells regarding PEMs in complex cellular scenarios is poorly understood. Thus, the objective of the present study was to investigate the interaction of PEMs with TCCs and, in particular, to probe for their transfer across the lung barrier from macrophages to dendritic cells located underneath the epithelium. Such possible cell-to-cell transfer of PEMs could potentially be exploited for inhalation-based applications targeting dendritic cells, which are numerously distributed in the respiratory tract and the key antigen-presenting cells, orchestrating both innate and adaptive immune functions [1]. PEMs composed of biodegradable walls have been used to release pro-drug molecules inside cells, which are activated only after cellular internalization [20]. Due to the time delay of activation after incorporation, it might be possible that PEMs first are internalized by macrophages, and only after having been passed to dendritic cells is their cargo activated, which would allow for specific targeting. Whether such concepts can be realized however will strongly depend on the capability to transfer PEMs between macrophages and dendritic cells, which is the topic of the present study.

## 2. Experimental details

### 2.1. Synthesis of PEMs

#### 2.1.1. Materials

Non-biodegradable PEMs made of poly(sodium 4-styrenesulfonate) (PSS) and poly(allylamine hydrochloride) (PAH), and biodegradable PEMs made of dextran sulfate (DextS) and poly-L-arginine (PLArg) were prepared as previously described [17, 20]. All chemicals used for PEM synthesis were obtained from Sigma Aldrich (USA) except fluorescein isothiocyanate (FITC)-dextran 500 kDa and dequenching DQ-OVA (Life Technologies, USA).

#### 2.1.2. Template core preparation

Either FITC-dextran or DQ-OVA were embedded into CaCO_3_ template microparticles by co-precipitation. In the case of FITC-loaded particles, 10 mL of aqueous solution of CaCl_2_ (0.33 M) and 10 mL FITC-dextran 500 kDa (0.25 mg mL^−1^) were mixed in a glass via l. During magnetic stirring (1000 rpm), 3 mL of aqueous solution of Na_2_CO_3_ (0.33 M) was added quickly. After 30 s, the solution was transferred into two 15 mL centrifuge tubes and after 2 min, the particle growth was terminated by centrifugation. The particles were washed three times with double distilled water. In the case of QD-OVA, filled CaCO_3_ template microparticles were synthesized using the same procedures, but with smaller amounts of materials: 615 *μ*L of CaCl_2_ (0.33 M) solution, 770 *μ*L DQ-OVA (50 *μ*M), and 615 *μ*L Na_2_CO_3_ (0.33 M), but elsewise the same procedures [21, 22].

#### 2.1.3. Biodegradable capsules

Layer-by-layer (LbL) assembly was carried out by adsorbing alternating layers of negatively charged DextS (M_w_ ≈ 40 kDa, 2 mg/mL in 0.5 M NaCl) and positively charged PLArg (M_w_ ≈ 15–70 kDa, 1 mg/mL in 0.5 M NaCl) onto the template cores. For each coating step, the microparticles were suspended in 1–5 mL of polyelectrolyte solution, followed by three washing steps (centrifugation in ddH_2_O). Finally, hollow FITC-dextran or DQ-OVA filled PEMs were obtained by dissolution of the CaCO_3_ templates by Ca^2+^ ion complexion with ethylenediaminetetraacetic acid (EDTA, 0.2 M, pH 7) [17, 20].

#### 2.1.4. Non-biodegradable capsules

Synthesis was performed analogously to that of the biodegradable PEMs with PSS (M_w_ ≈ 70 kDa) instead of DextS and with PAH (M_w_ ≈ 56 kDa) instead of PLArg [17, 20]. A summary of the prepared PEMs with some of their physicochemical parameters is given in the Supporting Information (Supporting Information, figure 1).

**Figure 1.**
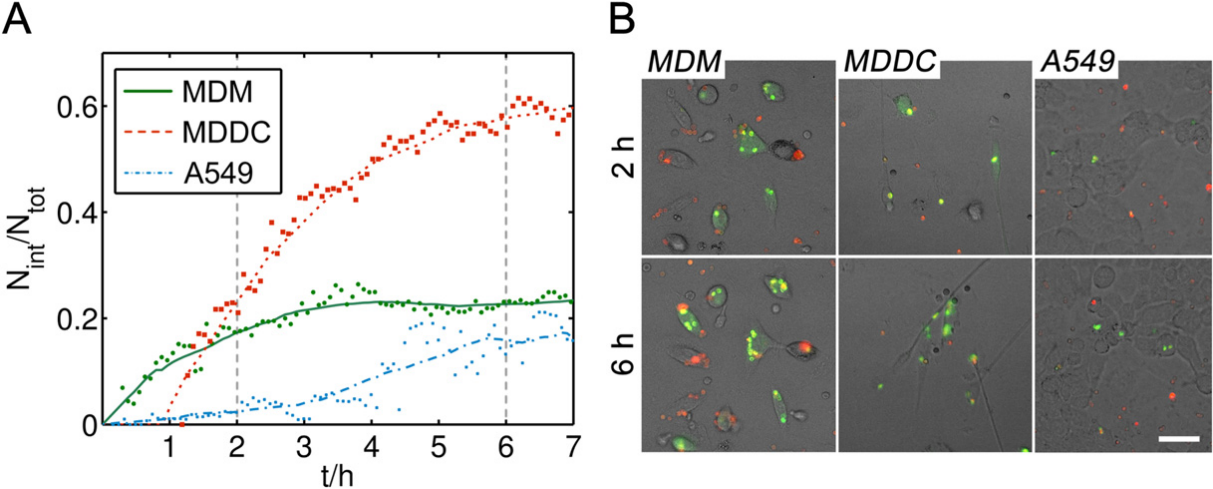
(A) Kinetics of PEM uptake for the different types of cells used for the experiments. Cells were exposed to DextS/PLArg PEMs filled with BODIPY-labeled DQ-OVA. By investigating the color change from red to green emission due to enzymatic digestion upon uptake, the fraction of internalized PEMs *N*_int_/*N*_tot_ over time was quantified, where *N*_int_ represents the number of internalized and *N*_tot_ represents the total number of PEMs visible in a randomly chosen area. (B) Representative fluorescence micrographs are shown for two time points (2 h and 6 h). Non-internalized PEMs appear red. Green fluorescence is associated with digested and released DQ-OVA. The scale bar corresponds to 50 *μ*m.

### 2.2. Cell culture techniques

#### 2.2.1. Culture of cell lines

Human alveolar epithelial type II cells (A549) from American Tissue Type Culture Collection (ATTC #CCL-185) were cultured in Roswell Park Memorial Institute Medium (RPMI 1640, Gibco, Luzern, Switzerland) with 4-(2-hydroxyethyl)-1-piperazineethanesulfonic acid (HEPES, Gibco) supplemented with 10% fetal bovine serum (FBS, heat inactivated, PAA Laboratories, Austria), 1% L-glutamine (L-glut, Gibco, Luzern, Switzerland), and 1% penicillin/streptomycin (Gibco, Luzern, Switzerland) and maintained at 37 °C and 5% CO_2_. A549 epithelial cells were sub-cultured twice per week using trypsin (0.05% trypsin-EDTA, Gibco). Five days prior to TCC preparation, A549 cells were seeded at a density of 2 × 10^5^ cells/mL in the upper chamber of membrane inserts (BD Falcon, 0.3 cm^2^ surface area, 3.0 *μ*m pores, transparent PET-membrane), placed into growth medium containing 24 well plates (Milian, Satigny, Switzerland), and grown to confluence. The growth medium was changed once before TCC preparation.

#### 2.2.2. Isolation and monocyte differentiation

Monocytes were isolated from buffy coats from healthy donors (blood donation service, Bern, Switzerland) as previously described [4]. For differentiation into MDDCs, monocytes were cultured in RPMI 1640 medium supplemented with 10% FBS (heat inactivated, PAA Laboratories, Austria), 1% L-glut (Gibco, Luzern, Switzerland), 1% penicillin/streptomycin (Gibco, Luzern, Switzerland), 10 ng mL^−1^ granulocyte macrophage colony-stimulating factor (GM-CSF, Miltenyi Biotech, Bergisch Gladbach, Germany), and 10 ng mL^−1^ interleukin-4 (IL-4, Miltenyi Biotech, Bergisch Gladbach, Germany). For differentiation into MDMs, no growth factors were added. Differentiation was performed in six well plates (10^6^ cells/mL; 3 mL/well supplemented growth medium) for 6 days at 37 °C and 5% CO_2_. Differentiation and maturation into MDDCs and MDMs applying this culture conditions have been demonstrated in earlier studies [23, 24]. For the experiments, the primary cells were harvested after differentiation by off-scraping.

#### 2.2.3. Cell preparation for live cell imaging

1.2 × 10^5^ MDMs/mL, 0.8 × 10^5^ MDDCs/mL, or 2 × 10^5^ A549 cells/mL, respectively, were grown inside 35 mm *μ*-dishes (Ibidi). After 24 h, the cells were placed inside an environmental module at 5% CO_2_ and 37 °C, DQ-OVA filled PEMs were added (15 PEMs/cell), and live imaging was performed.

#### 2.2.4. Cell preparation for live cell imaging of bi-cultures

1.2 × 10^5^ MDMs/mL were grown inside 35 mm *μ*-dishes (Ibidi) and loaded with PEMs (15 PEMs/cell) for 2 h. Afterwards, 0.8 × 10^5^ MDDCs/mL were added, which had been stained with tetramethylrhodamine-labeled wheat germ agglutinin (WGA, Life Technologies).

#### 2.2.5. Cell preparation for live cell imaging of TCCs

A549 cells were fluorescently stained with CellTracker violet BMQC dye at a dilution of 1:1000 for 1 h at 37 °C followed by three washing steps with phosphate buffered saline (PBS). Simultaneously staining of mature MDDCs was performed inside the chambers of the six well plates that were used for differentiation. After triple washing, off scraping and counting, MDDCs were added to the membrane inserts (8.9 × 10^3^ cm^−2^), containing stained A549 cells on the top side, to adhere at the bottom side by turning the insert upside down for 1 h. During the attachment process, MDMs were stained in the same way as MDDCs. Finally, 3.5 × 10^3^ MDMs mL^−1^ were added on top of the A549 cells. PEMs were added into the upper well prior to image acquisition by confocal laser scanning microscopy (CLSM) and tracked over 24 h. In another approach, MDMs were pre-incubated with PEMs for 24 h and then washed three times, scratched off, and added to A549 cells and MDDCs already being attached to the insert.

#### 2.2.6. Cell preparation for flow cytometry (FCM) measurements

MDMs were exposed to non-biodegradable PEMs (5 PEMs/cell) for 24 h. Afterwards, MDMs were washed three times, scraped off, re-suspended in fresh medium, and spun down for 3 min at 500 rcf. Simultaneously, MDDCs were prepared for bi-culture. MDMs and MDDCs (1.25 × 10^6^ cells mL^−1^; 1:1 ratio), respectively, were co-exposed in a new chamber of a six well plate another for 24 h for possible cell-to-cell PEM transfer to take place. On day 7, the cells were washed three times, scraped off, re-suspended in PBS, and spun down for 3 min at 500 rcf. The supernatant was replaced by fragment crystallizable blocking receptor reagent (FCM-Block, Miltenyi Biotech #130059901) for 10 min in order to reduce nonspecific binding. Then, cells were re-suspended in 1 mL of FCM buffer (1% bovine serum albumin (Sigma Aldrich, St. Louis, MO, USA) and 0.1% NaN_3_ (Sigma Aldrich, St. Louis, MO, USA) in PBS, (Life technologies, CA, USA) and stained with the following antibodies: CD1c-Pacific Blue (Biolegend #331507) for MDDCs and CD14-Brilliant Violet (Biolegend #301830) for MDMs. Finally, cells were washed and FCM analysis was performed.

### 2.3. Analysis techniques

#### 2.3.1. Confocal fluorescence microscopy

Cells were kept in an environmental module at 5% CO_2_ and 37 °C. Image acquisition was carried out with a CLSM 710 Meta setup (Zeiss, Germany/Switzerland) equipped with lasers for excitation at 405 nm, 488 nm, or 543 nm. In the case of observing the individual cells types separately for obtaining the kinetics of PEM uptake, images were acquired at a temporal resolution of 5 min using a Plan-Apochromat 20×/0.8 M27 objective. Fluorescence of DQ-OVA was excited at 488 nm and 543 nm and the emission was collected between 500–540 nm (green channel) and 560–750 nm (red channel). PEMs were counted based on the time-lapse image series using CellProfiler [25], whereby internalized PEMs were identified by their increased fluorescence in the green channel upon contact with digestive enzymes after engulfment. In the case of bi-cultures and TCCs, analysis was performed using a 25x objective with a numerical aperture of 1.4 and an immersion oil lens. After TCC imaging, cellular and morphological information was retrieved using Imaris (Bitplane 7.4, Zürich, Switzerland). The spots module was applied and set to 5 *μ*m in order to track the PEMs throughout the TCCs. Further, the cells morphology was restored in 3D by surface rendering of smoothed data using the same software.

#### 2.3.2. Flow cytometry

FCM was carried out with a BD-LSR Fortessa machine. The entire procedure was performed on ice and 10 000 events were recorded. The obtained data were analyzed with FlowJo (Tree Star). Representative gating strategies are shown in the Supporting Information.

## 3. Results

### 3.1. Uptake of biodegradable capsules

In order to investigate whether PEMs or fragments of digested PEMs can be passed from MDMs to MDDCs, DQ-OVA labeled with boron-dipyrromethene (BODIPY) was encapsulated into biodegradable DextS/PLArg PEMs as described previously [20]. The green fluorescence of the dye BODIPY is almost completely self-quenched due to the close proximity of the dye molecules inside non-fragmented ovalbumin [26]. At high dye concentrations, as occurring inside PEMs, slightly red fluorescent excimers are formed. After enzymatic degradation during uptake by cells, the green fluorescence intensity of BODIPY is dramatically increased and internalized PEMs and potentially released and passed fragments of DQ-OVA become visible [20].

For recording the uptake kinetics of PEMs in MDMs, MDDCs, and A549 epithelial cells, all types of cells were exposed to DQ-OVA filled PEMs and the ratio *N*_int_/*N*_tot_ of internalized to total PEMs was calculated over time. Hereby, *N*_tot_ refers to the total number of capsules and *N*_int_ to the number of internalized PEMs present in a randomly chosen area recorded by CLSM (size of recorded areas ≈0.2 mm^2^). The change in color from red to green fluorescence of BODIPY-labeled DQ-OVA allowed for distinguishing between internalized PEMs and PEMs outside cells (figure 1(B)). MDMs showed the faster kinetics of internalization (green curve in figure 1(A)), followed by MDDCs (red curve in figure 1(A)), and A549 cells (blue curve in figure 1(A)). In contrast to MDMs, MDDCs are highly mobile. Thus, they were able to take up much more PEMs present over time, whereas MDMs could only reach and internalize PEMs in close proximity and a certain fraction of non-internalized PEMs always remained, depending on the cell density.

**Figure 2.**
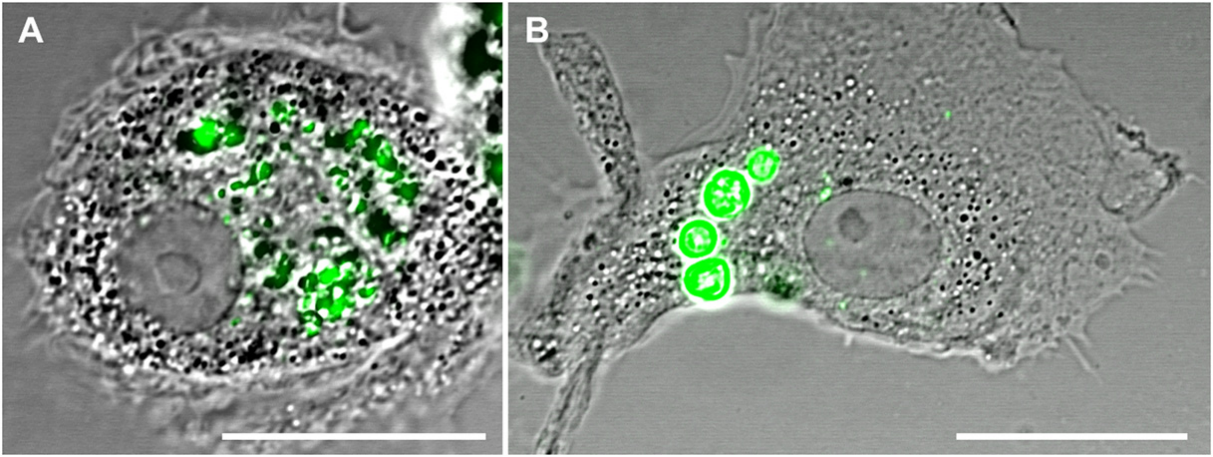
Biodegradable PEMs (A) and non-biodegradable PEMs (B) engulfed by MDMs at 1 h of exposure. Cells are shown in differential interference contrast (DIC; grey) and PEMs are shown in green. The scale bar corresponds to 20 *μ*m. More images are shown in figure SI-2.

**Figure 3.**
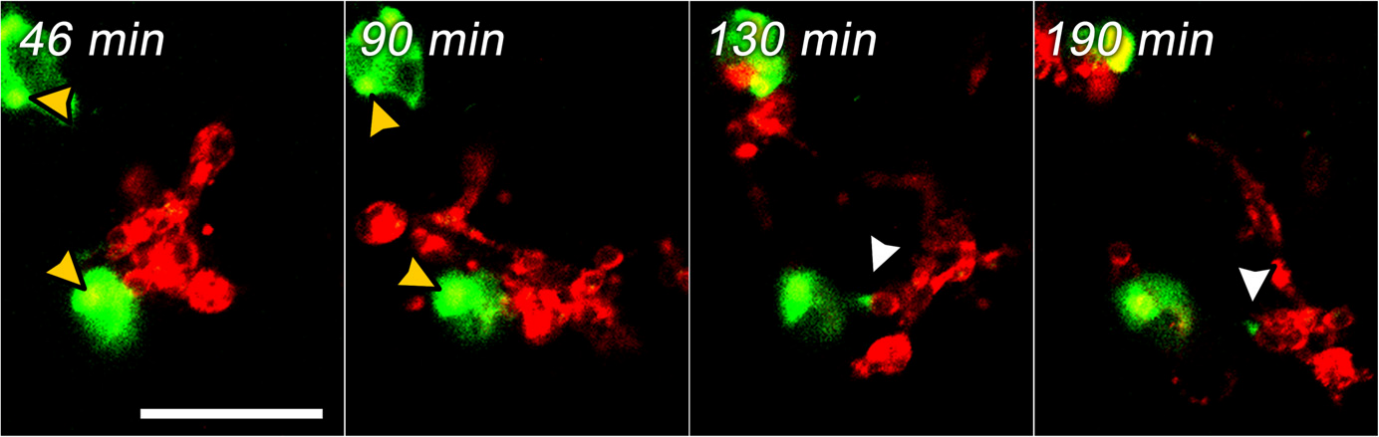
Possible MDM-to-MDDC transfer of PEM-released BODIPY-labeled DQ-OVA fragments in MDM/MDDC bi-culture. MDMs (appearing in green due to engulfed PEMs) were loaded with biodegradable DextS/PLArg PEMs filled with BODIPY-labeled DQ-OVA for 2 h. Subsequently, MDDCs marked with fluorescently labeled WGA (red) were added. PEMs appear as yellow spots, while digested DQ-OVA exhibits bright green fluorescence. The transfer of released DQ-OVA is marked with white arrows. The scale bar corresponds to 50 *μ*m.

Immediately after uptake, intracellular degradation of encapsulated DQ-OVA was observable by a partial release of BODIPY dye into the cytosol of both, MDMs and MDDCs (figure 1). In A549 cells, only degradation inside the cavities of the capsules but no release of encapsulated material was visible. Within 24 h, fragmentation of the capsules could be already detected in MDMs, as shown by CLSM (figure 2). In bi-cultures of capsule-loaded MDMs and MDDCs, one possible transfer of PEM-released BODIPY-labeled DQ-OVA fragments from one MDM to a MDDC could be witnessed (figure 3) during excessive imaging.

**Figure 4.**
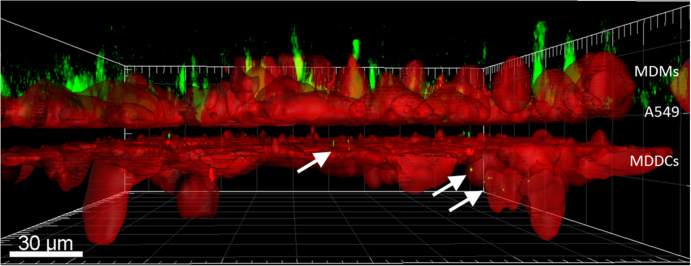
Lateral view of a TCC model exposed to biodegradable DexS/PLArg PEMs containing BODIPY-labeled DQ-OVA after 4.5 h of incubation (surface rendering). Capsules were added on top of the MDM cells and can be seen by the green fluorescence of DQ-OVA. Fluorescence signals of BODIPY (green) on the bottom side of the insert are highlighted by white arrows. Cellular plasma membranes are shown in red. The dark gap between the two red cell layers represents the membrane of the insert.

When TCCs composed of epithelial cells (A549), MDMs on top as well as MDDCs at the bottom of the membrane inserts were exposed to biodegradable DextS/PLArg capsules, PEMs were engulfed by MDMs in a time frame of 30 min and a partial release of DQ-OVA into the cytosol occurred similarly than observed in monocultures (figure 1). Some fluorescence signals of the degraded PEMs could be detected in MDDCs on the bottom side of the membrane (figure 4). Additionally, F-actin protrusions of MDDCs from the lower chamber have been observed to penetrate the pores of the insert membrane reaching the upper side of the chamber. During live cell imaging, the visualization of transfer events of released BODIPY-labeled DQ-OVA from MDMs to MDDCs was not possible because of the rather long time-intervals between acquisitions of the single 3D-stacks. In bi-cultures of MDMs and MDDCs, only one possible transfer event was observed during excessive imaging (figure 3, *t* = 130 min). Thus, a direct passing of digested PEM fragments from MDMs to MDDCs in bi-cultures as well as in TCCs was difficult to track and almost not observable by CLSM within several time-lapse recordings of live cells.

### 3.2. Uptake of non-biodegradable capsules

**Figure 5.**
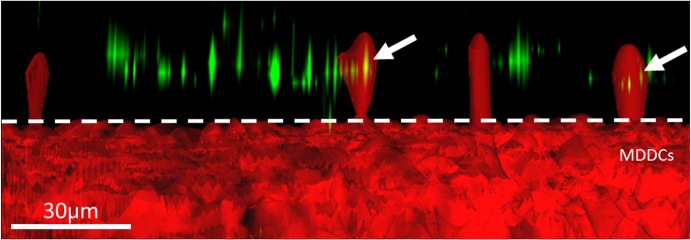
Side view of a TCC (3D surface rendering) exposed to non-biodegradable PSS/PAH PEMs filled with BODIPY-labeled DQ-OVA (green) after 1 h. For clarification, only MDDCs were fluorescently stained (red). White arrows indicate F-actin protrusions of MDDCs through the pores of the membrane insert (white dotted line). A549 and MDMs are not visible in this representation.

Instead of imaging the cargo (BODIPY) of biodegradable capsules as described previously, we probed the possible detection of full PEM transfer events. For this purpose, cells exposed to non-degradable PEMs based on PSS/PAH were recorded in both CLSM and high content screening experiments realized with FCM. Uptake of PEMs in TCCs was analyzed with CLSM first. Hereby, the PEMs were added on top of the membrane inserts, i.e., to the upper chamber in which the MDMs were grown. As CLSM allows for lateral resolution (in contrast to FCM), these measurements can help us retrieve more details about the F-actin protrusions being continuously formed by MDDCs grown in the lower chamber toward the upper chamber of the inserts. In contrast to data shown in figure 4, in this experiment, only MDDCs were stained with CellTracker violet BMQC dye (figure 5).

Again, persistent ‘grabbing’ of MDDCs for the PEMs was observed, but no PEMs were ‘pulled down’, i.e., no transfer of PEMs from the upper to the lower chamber could be observed. F-actin protrusions formed by MDDCs occurred similarly as observed in the previous exposure experiments with biodegradable PEMs. Also, in case MDMs were pre-incubated with PEMs for 24 h before adding them to the bi-culture system, the PEMs were not detected in MDDCs. Thus, CLSM data did not indicate transfer of PEMs from MDMs to MDDCs across the epithelial barrier formed by A549 cells.

**Figure 6.**
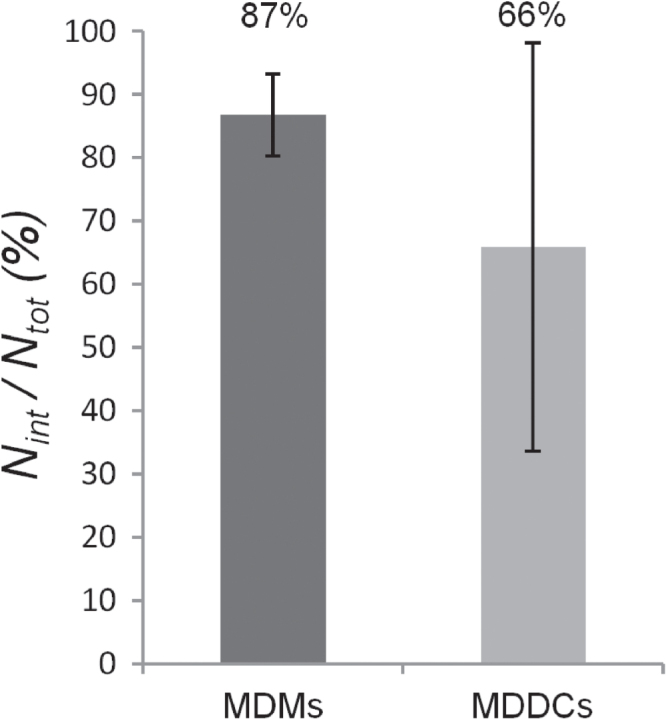
Investigation of PEM transfer events within 24 h from initially PEM-loaded MDMs to MDDCs in bi-culture, as observed by FCM. The *y*-axis represents the frequency *N*_int_/*N*_tot_ (%) of MDMs and MDDCs having internalized non-degradable PEMs. Hereby, *N*_int_ corresponds to the number of cells with internalized PEMs and *N*_tot_ to the total number of cells (the gating strategy can be found in the Supporting Information, figure SI-3). MDMs were marked with CD14 and MDDCs with CD1c antibodies. Experiments were performed in triplicates and results are given as mean ± standard deviation.

As ‘grabbing’ of MDDCs towards PEM-loaded MDMs was observed in the TCC model, possible PEM transfer events were also investigated by FCM within bi-cultures of MDMs and MDMs. MDMs were pre-loaded with non-biodegradable PSS/PAH PEMs filled with fluorescein isothiocyanate (FITC) and added to cultures of MDDCs. After 24 h, cells were analyzed by FCM (figure 6). In 87% of all MDMs, fluorescence signals of the PEMs could be found and 66% of all MDDCs were fluorescent after 24 h of co-culturing. Comparing the two primary cell types, the standard deviation for MDDCs is much larger than the standard deviation for MDMs (figure 6).

## 4. Discussion

To investigate uptake and possible cell-to-cell transfer of released material from PEMs or whole PEM transfer events between two different types of immune cells, i.e., from MDMs to MDDCs, two different types of PEMs were used. Biodegradable PEMs were prepared to trace released fluorescent molecules, whereas non-biodegradable PEMs seemed reasonable to trace whole PEM cell-to-cell transfer events acting as control in case transfer of released material from biodegradable PEMs would have occurred in high frequency. Qualitative CLSM, as well as quantitative FCM analysis, was applied. The application of live cell imaging for visualization of TCCs is very challenging, since the thickness of the whole tissue barrier is about 50–80 *μ*m. In addition, MDDCs are highly motile and thus, live imaging requires a high temporal resolution to resolve cell-to-cell interactions. In this case, image acquisition cannot be realized by conventional CLSM, but would need spinning-disc techniques. However, with our setup, we could show that the process of uptake and ingestion of biodegradable PEMs by MDMs was very fast, i.e., within 30–45 min, as shown by others [27]. In experiments with TCCs, signals of biodegradable PEMs were detected inside MDDCs on the bottom side of the chamber after 4.5 h. Capsule fragments were possibly transferred directly from MDMs to MDDCs via tight junctions through the epithelium. This process has been observed for polystyrene particles with 1 *μ*m diameter in case of MDM/MDDC co-cultures by Blank *et al* [4, 5]. However, since intracellular release of DQ-OVA from PEMs in MDMs starts taking place immediately after uptake (figure 1), it could also be possible that MDDCs came into contact with exocytozed PEM fragments. These fragments might have been secreted by either MDMs or A549 cells into the surrounding medium or could have been directly transmitted from MDMs/A549 cells to MDDCs. Another possibility could be that the released PEM fragments from the MDMs have passed through the 3 *μ*m-sized membrane pores (either directly or via A549 cells) followed by subsequent active uptake by MDDCs. Even internalized but non-fragmented capsules should be able to pass the pores of the membrane once internalized, although they are larger in size: They are rather elastic (mechanical stiffness <2 Nm^−1^) [27] and, according to previous studies, can be deformed and compressed by cells easily [15, 27, 28]. As the capsules are slightly larger than the pore size, passive diffusion from the top to the bottom side of the insert can almost be excluded. Hence, capsules being present on the MDDC side must have actively been transferred by cells.

In order to confirm the exact route of the released capsules/capsule fragments from MDMs toward the bottom side of the membrane insert in the two-chamber system, it would be necessary to completed more observations with microscopy techniques that allow a higher temporal resolution. The non-biodegradable PEMs were not transmitted to MDDCs residing at the bottom side of the insert under any conditions. We assume that this is a matter of size, since it was shown that 1 *μ*m polystyrene beads were detected inside MDDCs on the bottom side of the insert in TCCs after initial exposure of MDMs with the particles [4, 24, 29]. For both architectures of PEMs, cellular F-actin protrusion from MDDCs was detected throughout the exposure. These protrusions were well visualized when applying image restoration. It is known that F-actin filament growth occurs within microseconds [29] and is a prerequisite for cells to move, grow, scan, and sense their surrounding environment [30, 31]. Since F-actin protrusions were observed to occur throughout the measurement, the cells seemed to try to reach out for capsules persistently without any success.

Whereas live cell imaging allows for the analysis of entire TCCs, in additionally performed FCM measurements, only bi-cultures were examined. Although the two approaches of live cell imaging and FCM differed in their sample preparation and detection, the findings coincided in terms of no observed capsule transmission from MDMs to MDDCs. In a supplementary performed FCM-based control experiment, capsule transmission from MDMs to epithelial A549 cells, instead of MDDCs, was probed. Similar results were observed, i.e., capsules could be detected inside A549 cells with the same frequency as inside MDDCs; hence, FCM experiments could neither verify PEM transfer from MDMs to MDDCs. This indicates that in these bi-cultures, PEM uptake most probably derived from the free PEMs in the surrounding medium. Although validation is required, non-phagocytic A549 cells seemed to be a good control, since their function as barrier forming cells is far different from the function of immune cells, which perform phagocytosis and crosstalk with other cells. Thus, based on the FCM data, we could not confirm any cell-to-cell transfer of PEMs between MDMs and MDDCs.

## References

[C1] von Garnier C, Rothen-Rutishauser B, Blank F (2013). Nanoparticles in the respiratory tract: modulation of antigen-presenting cell function. J. Environ. Immunol. Toxicol..

[C2] Müller L, Lehmann A D, Johnston B D, Blank F, Wick P, Fink A, Rothen-Rutishauser B, Sahu S C, Casciano D A (2014). Inhalation pathway as a promising portal of entry: what has to be considered in designing new nanomaterials for biomedical application?. Handbook of Nanotoxicology, Nanomedicine and Stem Cell Use in Toxicology.

[C3] Rothen-Rutishauser B, Blank F, Mühlfeld C, Gehr P (2009). Nanoparticle-cell membrane interactions. Particle–Lung Interactions.

[C4] Blank F, Wehrli M, Lehmann A, Baum O, Gehr P, von Garnier C, Rothen-Rutishauser B M (2011). Macrophages and dendritic cells express tight junction proteins and exchange particles in an *in vitro* model of the human airway wall. Immunobiol.

[C5] Blank F, Rothen-Rutishauser B, Gehr P (2007). Dendritic cells and macrophages form a transepithelial network against foreign particulate antigens. Amer. J. Resp. Cell Mol. Bio..

[C6] Donath E, Sukhorukov G B, Caruso F, Davis S A, Möhwald H (1998). Novel hollow polymer shells by colloid-templated assembly of polyelectrolytes. Angew. Chem. Int. Edn.

[C7] Rivera Gil P, del Mercato L L, del Pino P, Muñoz-Javier A, Parak W J (2008). Nanoparticle-modified polyelectrolyte capsules. Nano Today.

[C8] De Geest B G, De Koker S, Sukhorukov G B, Kreft O, Parak W J, Skirtach A G, Demeester J, De Smedt S C, Hennink W E (2009). Polyelectrolyte microcapsules for biomedical applications. Soft Matter.

[C9] Borges J, Mano J F (2014). Molecular interactions driving the layer-by-layer assembly of multilayers. Chem. Rev..

[C10] del Mercato L L, Rivera Gil P, Abbasi A Z, Ochs M, Ganas C, Zins I, Sönnichsen C, Parak W J (2010). Lbl multilayer capsules: recent progress and future outlook for their use in life sciences. Nanoscale.

[C11] Rivera Gil P, Parak W J (2008). Composite nanoparticles take aim at cancer. ACS Nano.

[C12] Muñoz Javier A, del Pino P, Bedard M F, Skirtach A G, Ho D, Sukhorukov G B, Plank C, Parak W J (2008). Photoactivated release of cargo from the cavity of polyelectrolyte capsules to the cytosol of cells. Langmuir.

[C13] Bedard M F, Munoz-Javier A, Mueller R, del Pino P, Fery A, Parak W J, Skirtach A G, Sukhorukov G B (2009). On the mechanical stability of polymeric microcontainers functionalized with nanoparticles. Soft Matter.

[C14] Ganas C, Weiß A, Nazarenus M, Rösler S, Kissel T, Rivera Gil P, Parak W J (2014). Biodegradable capsules as non-viral vectors for *in vitro* delivery of pei/sirna polyplexes for efficient gene silencing. J. Control Release.

[C15] Muñoz_Javier A (2008). Uptake of colloidal polyelectrolyte coated particles and polyelectrolyte multilayer capsules by living cells. Adv. Mat..

[C16] Reibetanz U, Halozan D, Brumen M, Donath E (2007). Flow cytometry of Hek 293 t cells interacting with polyelectrolyte multilayer capsules containing fluorescein-labeled poly(acrylic acid) as a pH sensor. Biomacromolecules.

[C17] Kastl L (2013). Multiple internalization pathways of polyelectrolyte multilayer capsules into mammalian cells. ACS Nano.

[C18] Kirchner C, Javier A M, Susha A S, Rogach A L, Kreft O, Sukhorukov G B, Parak W J (2005). Cytotoxicity of nanoparticle-loaded polymer capsules. Talanta.

[C19] De Geest B G (2012). Polymeric multilayer capsule-mediated vaccination induces protective immunity against cancer and viral infection. ACS Nano.

[C20] Rivera Gil P, Koker S D, De Geest B G, Parak W J (2009). Intracellular processing of proteins mediated by biodegradable polyelectrolyte capsules. Nano Lett..

[C21] Sukhorukov G, Dähne L, Hartmann J, Doanth E, Möhwald H (2000). Controlled precipitation of dyes into hollow polyelectrolyte capsules based on colloids and biocolloids. Adv. Mat..

[C22] Antipov A A, Sukhorukov G B (2004). Polyelectrolyte multilayer capsules as vehicles with tunable permeability. Adv. Colloid Interface Sci..

[C23] Lehmann A, Brandenberger C, Blank F, Gehr P, Rothen-Rutishauser B (2010). A 3D model of the human epithelial airway barrier. Methods Bioengin: Alt Tech. Animal Testing.

[C24] Rothen-Rutishauser B M, Kiama S G, Gehr P (2005). A three-dimensional cellular model of the human respiratory tract to study the interaction with particles. Am. J. Resp. Cell Mol. Bio..

[C25] Carpenter A (2006). Cellprofiler: image analysis software for identifying and quantifying cell phenotypes. Genome Bio..

[C26] Mansour M K, Latz E, Levitz S M (2006). Cryptococcus neoformans glycoantigens are captured by multiple lectin receptors and presented by dendritic cells. J. Immunol..

[C27] Hartmann R, Weidenbach M, Neubauer M, Fery A, Parak W J (2015). Stiffness-dependent in vitro uptake and lysosomal acidification of colloidal particles. Angew. Chem. Int. Edn.

[C28] Palankar R, Pinchasik B E, Schmidt S, De Geest B G, Fery A, Möhwald H, Skirtach A, Delcea M (2013). Mechanical strength and intracellular uptake of caco 3-templated lbl capsules composed of biodegradable polyelectrolytes: the influence of the number of layers. J. Mater. Chem. B.

[C29] Rothen-Rutishauser B, Mühlfeld C, Blank F, Musso C, Gehr P (2007). Translocation of particles and inflammatory responses after exposure to fine particles and nanoparticles in an epithelial airway model. Part. Fibre Toxicol..

[C30] Kuhn J R, Pollard T D (2005). Real-time measurements of actin filament polymerization by total internal reflection fluorescence microscopy. Bio. J..

[C31] Ananthakrishnan R, Ehrlicher A (2007). The forces behind cell movement. Int. J. Bio. Sci..

